# Associação da Preservação Cultural Germânica Com o Risco Cardiovascular e a Aterosclerose Subclínica em Adultos do Sul do Brasil

**DOI:** 10.36660/abc.20260095

**Published:** 2026-11-04

**Authors:** Ribanna Aparecida Marques Braga, Marcello Ricardo Paulista Markus, Marcus Dörr, Henry Völzke, Martin Bahls, Siegmar Starke, Sergio Luiz Zimmermann, Ernani Tiaraju de Santa Helena, Raul D. Santos, Luiz Aparecido Bortolotto, Joany Coronado, Nagila Raquel Teixeira Damasceno

**Affiliations:** 1 Hospital das Clínicas Faculdade de Medicina Universidade de São Paulo São Paulo SP Brasil Instituto do Coração do Hospital das Clínicas da Faculdade de Medicina da Universidade de São Paulo, São Paulo, SP – Brasil; 2 University Medicine Greifswald Department and Polyclinic of Internal Medicine B Mecklenburg Vorpommern Alemanha University Medicine Greifswald Department and Polyclinic of Internal Medicine B - Medicine Greifswald, Mecklenburg-Vorpommern – Alemanha; 3 University Medicine Greifswald Institute of Community Medicine Mecklenburg Vorpommern Alemanha University Medicine Greifswald Institute of Community Medicine - Community Medicine Greifswald, Mecklenburg-Vorpommern – Alemanha; 4 Fundação Universidade Regional de Blumenau Blumenau SC Brasil Fundação Universidade Regional de Blumenau, Blumenau, SC – Brasil

**Keywords:** Fatores de Risco de Doenças Cardíacas, Hipertensão, Aterosclerose

## Abstract

**Fundamento:**

A migração envolve transições culturais e de estilo de vida que podem influenciar o risco de doença cardiovascular (DCV). A preservação e a adaptação culturais podem afetar a saúde cardiovascular por meio de mecanismos comportamentais e sociais.

**Objetivo:**

Avaliar a associação da preservação cultural germânica com o risco de DCV e a aterosclerose subclínica entre residentes de Pomerode, no Sul do Brasil, estratificados por faixa etária e sexo.

**Métodos:**

Este estudo transversal utilizou dados da linha de base, coletados entre 2014 e 2018, da coorte Vida e Saúde em Pomerode e incluiu 727 indivíduos com idade entre 20 e 79 anos e dados completos. A preservação cultural germânica foi definida como o hábito de falar alemão regularmente em casa e a participação em associações culturais germânicas. A aterosclerose subclínica foi definida pela presença de três ou mais placas carotídeas. O risco de DCV aterosclerótica em 10 anos foi estimado por meio das Equações de Coortes Agrupadas do American College of Cardiology e da American Heart Association. A significância estatística foi estabelecida em p < 0,05.

**Resultados:**

A média de idade foi de 43,7 anos, e 51,9% dos participantes eram mulheres. A preservação cultural germânica foi relatada por 65,61% dos participantes e foi mais frequente entre adultos mais velhos, indivíduos brancos, protestantes e indivíduos de nível socioeconômico médio (p < 0,05). Os participantes que relataram preservação cultural germânica também apresentaram menor comportamento sedentário, maior uso de medicamentos anti-hipertensivos e perfil cardiometabólico menos favorável. Nos modelos ajustados e estratificados por idade, a preservação cultural germânica esteve associada à hipertensão arterial sistêmica (HAS) (odds ratio [OR]: 2,79; IC 95%: 1,49-5,22) e à aterosclerose subclínica (OR: 3,01; IC 95%: 1,07-8,40) entre indivíduos com 50 anos ou mais. Após o ajuste para HAS, a associação com a aterosclerose subclínica foi atenuada e deixou de ser estatisticamente significativa.

**Conclusões:**

A preservação cultural germânica esteve associada à HAS e à aterosclerose subclínica entre adultos com 50 anos ou mais, ressaltando a importância de considerar o contexto cultural na caracterização do risco cardiovascular em populações em processo de envelhecimento.

## Graphical abstract

**Figura Central:**
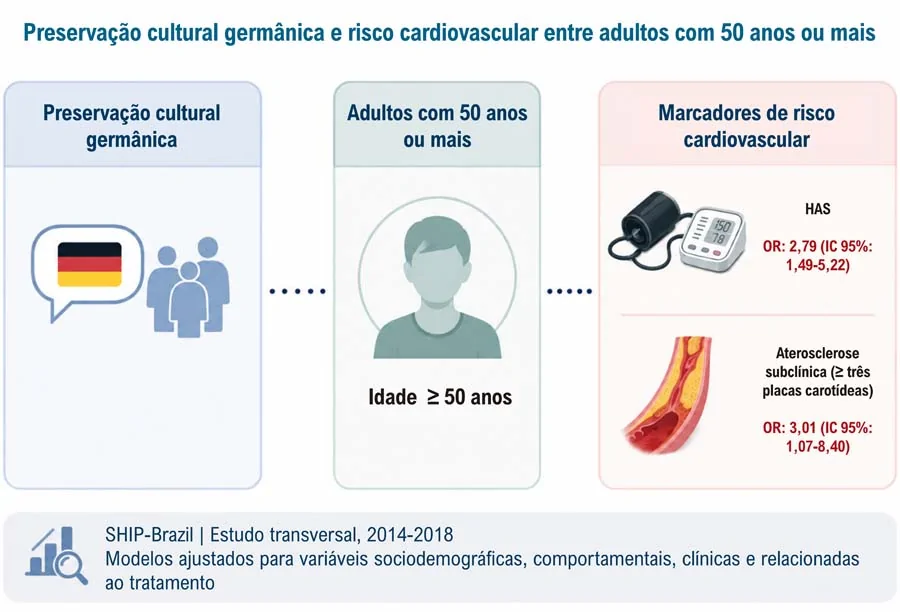
Associação da Preservação Cultural Germânica Com o Risco Cardiovascular e a Aterosclerose Subclínica em Adultos do Sul do Brasil

A preservação cultural germânica esteve associada a maiores chances de HAS e aterosclerose subclínica entre adultos com 50 anos ou mais. HAS: hipertensão arterial sistêmica; OR: odds ratio; SHIP-Brazil: Vida e Saúde em Pomerode.

## Introdução

A migração é um fenômeno humano de longa data que envolve mudanças culturais, sociais, econômicas e emocionais capazes de influenciar os desfechos de saúde.^1^ Independentemente de ocorrer dentro de um mesmo país ou entre países, a migração pode envolver diferentes graus de aculturação, caracterizados por mudanças nas práticas cotidianas, nos valores e na identidade, bem como pela preservação de tradições e comportamentos culturais ao longo das gerações.^2^ Esses processos culturais podem influenciar indicadores de saúde e perfis de risco cardiovascular.^3^

As diferenças na incidência, prevalência e mortalidade por doenças entre as populações migrantes e as populações dos países de acolhimento estão bem documentadas.^4,5^ Essas diferenças variam de acordo com o país de destino, a região de origem e a história migratória dos migrantes, bem como com o desfecho de doença cardiovascular (DCV) avaliado.^6^ Uma revisão sistemática identificou padrões heterogêneos de risco de DCV na Europa Ocidental e na América do Norte; por exemplo, migrantes oriundos do norte da África apresentaram menor risco de acidente vascular encefálico (AVE) do que a população do país de acolhimento nos Países Baixos.^5^ Os perfis de risco de DCV também diferem entre migrantes de primeira e segunda gerações e as populações dos países de acolhimento.^3-7^ Apesar dessa heterogeneidade, maiores prevalências de obesidade, diabetes tipo 2^8^ e hipertensão arterial sistêmica (HAS) têm sido consistentemente relatadas entre populações migrantes.^9^

A HAS, em particular, permanece como o fator de risco cardiovascular mais prevalente em todo o mundo^10,11^ e tem sido consistentemente associada a perfis cardiometabólicos desfavoráveis entre populações migrantes.^10-12^ No Brasil, a HAS continua sendo o principal fator de risco cardiovascular, afetando aproximadamente 30% da população.^13,14^ Entretanto, ainda são escassos os dados sobre o risco cardiovascular entre populações migrantes e seus descendentes no Brasil. Estudos brasileiros anteriores envolvendo populações migrantes, incluindo brasileiros de ascendência japonesa e migrantes africanos, demonstraram uma elevada carga de risco cardiometabólico.^15,16^ De modo semelhante, estudos realizados na Alemanha mostraram que, apesar do robusto sistema de saúde do país, a HAS e a dislipidemia continuam subdiagnosticadas e subtratadas, contribuindo para a persistência do risco cardiovascular.^17-21^ Contudo, a possível influência da preservação cultural germânica sobre os perfis de risco cardiovascular e a aterosclerose subclínica no Brasil permanece pouco compreendida.

Pomerode, localizada no Sul do Brasil, é conhecida por sua forte herança cultural germânica, com a preservação de seu idioma, suas tradições e suas práticas comunitárias ao longo das gerações. Esse contexto oferece uma oportunidade singular para examinar a relação entre a preservação cultural e a saúde cardiovascular em uma população com ascendência germânica de longa data. Portanto, este estudo investigou a associação da preservação cultural germânica com os perfis de risco cardiovascular e a aterosclerose subclínica entre adultos do Sul do Brasil, por meio de análises estratificadas por faixa etária e sexo.

## Métodos

### Delineamento do estudo e população

O presente estudo transversal utilizou dados da linha de base, coletados entre 2014 e 2018, da coorte Vida e Saúde em Pomerode (SHIP-Brazil). A estratégia de amostragem e os métodos empregados nessa coorte foram alinhados aos da coorte Study of Health in Pomerania (SHIP)-TREND.^22^ Os detalhes do delineamento do estudo, da estrutura conceitual e dos procedimentos foram publicados anteriormente.^23^

Uma amostra aleatória foi selecionada em 12 estratos definidos por sexo e idade e incluiu indivíduos com idade entre 20 e 79 anos. O tamanho amostral foi calculado considerando-se uma prevalência do desfecho de 50%, precisão de 5% e nível de confiança de 95%, resultando em uma amostra necessária de 3.678 indivíduos. Foram considerados elegíveis os indivíduos que residiam no município de Pomerode, no estado de Santa Catarina, Brasil, havia pelo menos 6 meses. Os critérios de exclusão foram: i) comprometimento físico ou cognitivo ou transtorno mental que impedisse o preenchimento dos questionários ou o comparecimento ao Centro de Exames do Hospital Universitário da Universidade Regional de Blumenau; e ii) incapacidade de falar português.

Após perdas e recusas de aproximadamente 30% durante a coleta de dados da linha de base, 2.488 indivíduos foram entrevistados. A análise incluiu uma subamostra de participantes que forneceram amostras biológicas e apresentavam dados completos para as variáveis de interesse. Entre os participantes entrevistados, 753 forneceram sangue e outras amostras biológicas. Posteriormente, foram excluídos 26 participantes sem informações sobre preservação cultural germânica, resultando em uma amostra analítica final de 727 participantes.

O estudo foi conduzido de acordo com os princípios éticos da Declaração de Helsinque. Todos os participantes assinaram termo de consentimento livre e esclarecido. O protocolo do estudo foi aprovado pelos comitês de ética em pesquisa sob os números de aprovação 99559118.0.0000.5370 e 40160420.5.0000.5421.

### Dados sociodemográficos e de estilo de vida

Os participantes foram entrevistados por meio de procedimentos padronizados e questionários estruturados. As variáveis sociodemográficas avaliadas incluíram: i) sexo (homens ou mulheres); ii) idade (contínua e categorizada como < 50 ou ≥ 50 anos); iii) raça e etnia autodeclaradas (branca ou não branca); iv) estado civil (casado, solteiro, separado ou viúvo); v) religião (protestante, católica, sem filiação religiosa ou outra religião); vi) escolaridade (ensino superior [≥ 12 anos], ensino médio [9-11 anos], ensino fundamental [5-8 anos] ou menos de 4 anos de ensino fundamental ou nenhuma escolaridade formal); e vii) nível socioeconômico. O nível socioeconômico foi classificado como alto (classe A), médio (classe B) ou baixo (classes C, D e E), de acordo com o Critério de Classificação Econômica Brasil.^24^

As variáveis relacionadas ao estilo de vida incluíram: i) risco de uso indevido de álcool, classificado como baixo ou elevado (moderado, alto ou grave);^25^ ii) atividade física, avaliada por meio do Questionário Internacional de Atividade Física e categorizada como caminhada, atividade física moderada ou vigorosa, ou comportamento sedentário;^26^ e iii) tabagismo, categorizado como nunca fumante, ex-fumante ou fumante atual. Também foram coletados dados sobre o uso de medicamentos anti-hipertensivos.

### Preservação da cultura germânica

Os participantes foram classificados como preservadores da cultura germânica quando relataram tanto falar alemão regularmente em casa quanto participar de associações comunitárias ou culturais germânicas que preservam costumes alemães no Brasil, incluindo vestimentas, músicas e alimentos tradicionais.^27^

### Dados antropométricos

O peso e a altura foram medidos em quilogramas e metros, respectivamente, e o índice de massa corporal (IMC) foi calculado dividindo-se o peso em kg pela altura em m^2^. O excesso de peso foi definido como IMC igual ou superior a 25 kg/m^2^ em adultos e igual ou superior a 28 kg/m^2^ em idosos, de acordo com os critérios da Organização Mundial da Saúde^28^ e da Organização Pan-Americana da Saúde,^29^ respectivamente.

A circunferência da cintura foi medida em centímetros e classificada como elevada quando igual ou superior a 94 cm nos homens e a 80 cm nas mulheres.^30^ O percentual de massa adiposa foi estimado por meio de análise de impedância bioelétrica tetrapolar (BodyStat MDD1500) e classificado como elevado quando igual ou superior a 25% nos homens e a 30% nas mulheres.^31^

### Análises bioquímicas

Amostras de sangue em jejum foram coletadas após um período de jejum de 10 a 12 horas, em tubos contendo ácido etilenodiaminotetracético (1,0 mg/mL; BD, Brasil). As concentrações plasmáticas de colesterol total, triglicerídeos e colesterol da lipoproteína de alta densidade (HDL-c) foram mensuradas por métodos padronizados em um sistema automatizado. O colesterol da lipoproteína de baixa densidade (LDL-c) foi calculado por meio da equação de Friedewald^32^ quando as concentrações de triglicerídeos eram inferiores a 400 mg/dL:


 LDL-c = (colesterol total - HDL-c) - triglicerídeos/5

O perfil lipídico foi classificado de acordo com a Diretriz Brasileira de Dislipidemias e Prevenção da Aterosclerose – 2025^33^ como colesterol total elevado (≥ 190 mg/dL), LDL-c elevado (≥ 160 mg/dL), HDL-c baixo (≤ 40 mg/dL nos homens e ≤ 50 mg/dL nas mulheres), colesterol não HDL elevado (≥ 160 mg/dL) ou triglicerídeos elevados (≥ 150 mg/dL).

A glicose plasmática foi mensurada por meio do reagente Glicose PAP Liquiform (Labtest Diagnóstica S.A., Lagoa Santa, MG, Brasil). Foram utilizados os pontos de corte de 100 mg/dL ou mais para pré-diabetes e de 126 mg/dL ou mais para diabetes tipo 2.^34^ Os níveis de pressão arterial (PA) foram classificados de acordo com as Diretrizes Brasileiras de Hipertensão Arterial – 2020.^35^ A PA sistólica (PAS) e a PA diastólica (PAD) foram aferidas três vezes com um esfigmomanômetro, após os participantes permanecerem sentados e em repouso durante 5 minutos. A média da segunda e da terceira aferições foi utilizada nas análises.

### Aterosclerose subclínica

As artérias carótidas extracranianas foram examinadas por ultrassonografia em modo B com um transdutor linear de banda larga operando a 13 MHz. A placa carotídea foi definida como: i) uma protrusão focal do complexo íntima-média para o lúmen vascular, acima da camada íntima-média circundante; ou ii) um aumento focal da ecogenicidade em corte longitudinal acompanhado por irregularidade da superfície luminal, mesmo na ausência de protrusão substancial para o lúmen.

Os exames foram realizados por examinadores treinados e certificados, que avaliaram separadamente cada segmento arterial em planos longitudinal e transversal, com atenção especial à identificação de placas ateroscleróticas. Os vasos foram examinados a partir de múltiplos ângulos de insonação, variando das posições mais anteriores às mais posteriores, a fim de melhorar a detecção de placas pequenas e excêntricas.

As artérias carótidas comuns, internas e externas e as bifurcações carotídeas foram examinadas sistematicamente em ambos os lados. A aterosclerose subclínica foi definida pela presença de três ou mais placas carotídeas, refletindo a carga de placas nas carótidas.

### Risco de DCV em 10 anos

O risco em 10 anos de DCV aterosclerótica maior, definida como infarto do miocárdio não fatal, morte por doença coronariana ou AVE fatal ou não fatal, foi estimado por meio das Equações de Coortes Agrupadas do American College of Cardiology (ACA) e da American Heart Association (AHA).^36^ Essas equações incorporam sexo, idade, raça, PAS, colesterol total, HDL-c, presença de diabetes, tratamento anti-hipertensivo e tabagismo. Para valores situados fora dos intervalos aceitos pelas equações, foi utilizado o valor permitido mais próximo. O risco estimado de DCV aterosclerótica em 10 anos foi classificado como baixo (< 5%), limítrofe (5% a < 7,5%), intermediário (7,5% a < 20%) ou alto (≥ 20%). Para as análises, os participantes foram posteriormente agrupados nas categorias de risco baixo ou limítrofe e risco intermediário ou alto.

### Análise estatística

Foi realizada uma análise *post hoc* do poder estatístico por meio do G*Power, versão 3.1.9.7,^37^ para regressão linear múltipla. Os parâmetros incluíram tamanho de efeito moderado (*f*^2^ = 0,15), nível α de 0,05, tamanho amostral de 727 participantes e quatro preditores. Um teste bicaudal resultou em poder estatístico de 1,00, indicando poder adequado para detectar um efeito moderado. A análise apresentou 722 graus de liberdade e valor crítico de *t* de 1,96.

Como as distribuições de idade e sexo da amostra analítica do SHIP-Brazil diferiram das observadas na população de Pomerode, foi empregada uma abordagem em duas etapas de ponderação pelo inverso da probabilidade para minimizar o possível viés de seleção e melhorar a representatividade populacional. Primeiramente, estimou-se a probabilidade de inclusão de cada participante na amostra analítica de acordo com características demográficas, incluindo idade e sexo. Em seguida, os pesos pelo inverso da probabilidade foram calculados como o inverso das probabilidades estimadas de seleção e incorporados a todas as análises. Essa abordagem atribuiu peso proporcionalmente maior aos indivíduos pertencentes a grupos sub-representados na amostra analítica, melhorando, assim, a comparabilidade entre a amostra analítica e a população de Pomerode.

Foram realizadas análises descritivas, com as variáveis contínuas apresentadas como médias e intervalos de confiança de 95% (IC 95%) e as variáveis categóricas como números e porcentagens. Testes de Wald foram utilizados para avaliar as associações da preservação cultural germânica com fatores de risco cardiovascular e aterosclerose subclínica com base na carga de placas carotídeas. Posteriormente, foram ajustados modelos de regressão multivariável, com ajuste para covariáveis selecionadas *a priori* de acordo com sua relevância epidemiológica e com as associações identificadas nas análises bivariadas (p < 0,05). O sexo foi mantido em todos os modelos ajustados devido à sua relevância estabelecida para o risco cardiovascular.

A regressão linear múltipla foi utilizada para avaliar a associação da preservação cultural germânica com fatores de risco cardiometabólico individuais, com estratificação por faixa etária e ajuste para sexo, raça e etnia, religião, nível socioeconômico, atividade física e uso de medicamentos anti-hipertensivos. Antes do ajuste dos modelos, foram avaliados os pressupostos de linearidade, distribuição normal dos resíduos, homocedasticidade, independência dos erros e ausência de multicolinearidade. Todos os pressupostos necessários para a regressão linear múltipla foram atendidos.

Também foram ajustados modelos de regressão logística binária multivariável para avaliar as associações da preservação cultural germânica com marcadores de risco cardiovascular e aterosclerose subclínica, estratificadas por faixa etária. As covariáveis foram selecionadas de acordo com critérios teóricos e estatísticos para minimizar a colinearidade e evitar o sobreajuste. O modelo de HAS foi ajustado para sexo, raça e etnia, religião, nível socioeconômico e atividade física, uma vez que o uso de medicamentos anti-hipertensivos já estava incluído na definição operacional de HAS. Para o risco estimado em 10 anos de doença cardiovascular aterosclerótica, a atividade física, a religião e o nível socioeconômico foram incluídos como covariáveis, uma vez que as demais variáveis eram componentes das equações de risco. O conjunto de variáveis de ajuste utilizado nos modelos de regressão linear múltipla foi mantido para os demais desfechos.

Todas as análises estatísticas foram realizadas nos programas Stata/SE, versão 17 (StataCorp LLC); GraphPad Prism, versão 8.0.1 (GraphPad Software); ou R, versão 4.4.1 (R Foundation for Statistical Computing), com os pacotes “table1” e “ggplot2”. A significância estatística foi definida como p bicaudal < 0,05.

## Resultados

A amostra analítica foi composta por 727 participantes (Figura 1), dos quais 51,92% eram mulheres. A média de idade foi de 43,74 anos (IC 95%: 43,51-43,96). A preservação cultural germânica foi identificada em 65,61% dos participantes (n não ponderado = 509) e foi mais frequente entre participantes mais velhos e aqueles que se autodeclararam brancos. No total, 61,36% (n não ponderado = 486) relataram falar alemão em casa, e 21,24% (n não ponderado = 176) relataram participar de associações comunitárias ou culturais germânicas. Entre os participantes classificados como preservadores da cultura germânica, 93,53% falavam alemão em casa e 32,38% participavam dessas associações. Esses participantes também apresentaram maior probabilidade de serem protestantes, pertencerem ao nível socioeconômico médio, relatarem menor comportamento sedentário e utilizarem medicamentos anti-hipertensivos (Tabela 1).

**Figura 1:**
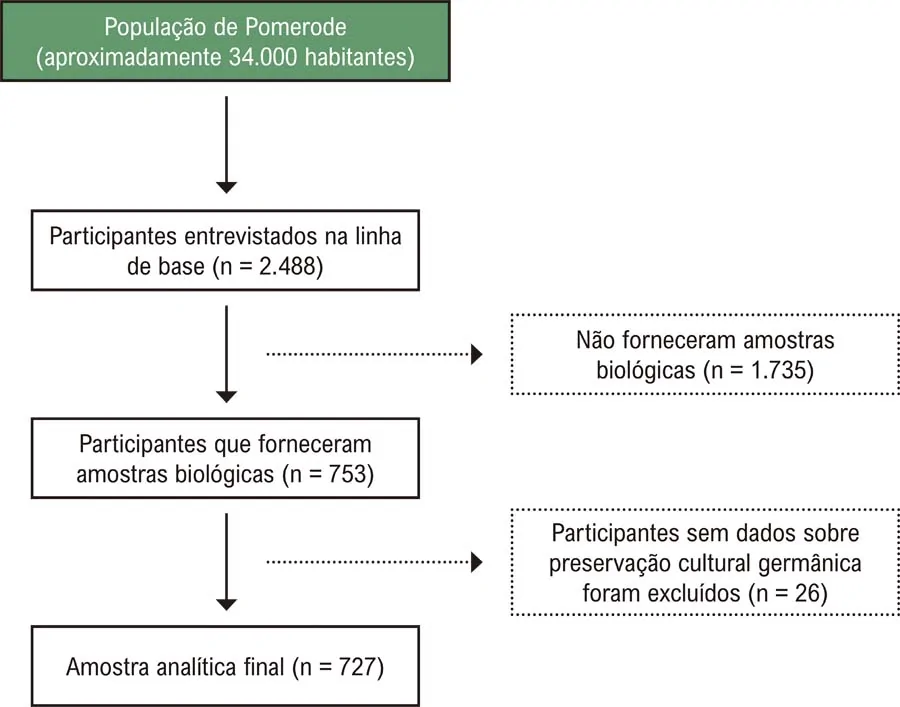
– Fluxograma da seleção da amostra do estudo e dos critérios de exclusão.

**Tabela 1:** – Características sociodemográficas e de estilo de vida segundo a preservação cultural germânica

Característica	Total, n	Total, % ponderada	Não, n	Não, % ponderada	Sim, n	Sim, % ponderada	Valor de p
**Sexo**							0,531
Homens	313	48,07	89	46,26	224	48,99	
Mulheres	414	51,92	129	53,73	285	51,00	
**Faixa etária, anos**							< 0,001
< 50	327	65,42	133	77,26	194	59,37	
≥ 50	400	34,57	85	22,73	315	40,62	
**Raça e etnia**							< 0,001
Não branca	41	5,66	34	16,71	7	2,45	
Branca	684	94,34	184	83,29	500	97,55	
Estado civil							0,251
Casado	549	71,44	167	72,92	382	70,66	
Solteiro	83	20,51	27	20,09	56	20,73	
Separado	30	3,53	14	4,50	16	3,02	
Viúvo	65	4,51	10	2,47	55	5,57	
**Religião**							0,014
Protestante	498	65,49	79	34,88	419	81,56	
Católica	204	29,79	126	57,60	78	15,20	
Sem filiação religiosa	12	2,26	7	3,38	5	1,67	
Outra	12	2,46	6	4,14	6	1,58	
**Escolaridade, anos**							0,062
Ensino superior (≥ 12)	87	18,95	32	18,47	55	19,20	
Ensino médio (9-11)	161	33,25	57	34,52	104	32,58	
Ensino fundamental (5-8)	145	18,94	69	29,54	76	13,35	
Ensino fundamental (< 4) ou nenhuma escolaridade	318	28,84	57	17,45	261	34,84	
**Nível socioeconômico**							0,002
Alto	116	17,58	25	11,99	91	20,52	
Médio	454	63,72	139	63,60	315	63,79	
Baixo	156	18,68	54	24,40	102	15,67	
**Risco de uso indevido de álcool**							0,194
Baixo	529	74,30	164	70,37	365	64,14	
Alto	183	25,70	54	29,63	129	35,86	
**Atividade física**							0,047
Caminhada ou atividade moderada/vigorosa	445	65,06	128	60,64	317	69,67	
Comportamento sedentário	239	34,94	80	39,36	159	30,33	
**Tabagismo**							0,391
Nunca fumante	499	69,60	149	71,24	350	75,83	
Ex-fumante	156	21,76	52	21,51	104	17,13	
Fumante atual	62	8,65	17	7,25	45	7,04	
**Uso de medicamentos anti-hipertensivos**							0,019
Sim	211	31,21	43	15,82	168	23,98	

Os dados são apresentados como frequências absolutas (n) e porcentagens ponderadas. As porcentagens foram calculadas utilizando pesos do plano amostral complexo e, portanto, podem diferir das proporções calculadas diretamente a partir das frequências observadas. A significância estatística foi definida como p < 0,05 pelo teste de Wald.

Conforme apresentado na Tabela 2, a preservação cultural germânica esteve associada a maiores médias de peso, circunferência da cintura e PAS (todos p < 0,005). Em consonância com esses achados, a HAS foi mais prevalente entre os participantes que preservavam a cultura germânica do que entre aqueles que não a preservavam (33,81% vs 20,80%). O excesso de peso apresentou elevada prevalência na amostra total (68,44%), e 37,59% dos participantes tinham obesidade. A prevalência de obesidade foi de 39,77% entre os participantes que preservavam a cultura germânica (p = 0,177; dados não apresentados). Os participantes que preservavam a cultura germânica também apresentaram maior risco estimado de DCV e maior prevalência de aterosclerose subclínica (Tabela 3).

**Tabela 2:** – Características cardiometabólicas segundo a preservação cultural germânica na coorte SHIP-Brazil, 2014-2018

Característica cardiometabólica	Total (n = 727), média	IC 95%	Não (n = 218), média	IC 95%	Sim (n = 509), média	IC 95%	Valor de p
Peso, kg	81,07	79,64-82,51	78,58	75,68-81,48	82,69	80,73-84,65	0,027
IMC, kg/m^2^	28,91	28,45-29,37	28,59	27,73-29,45	29,01	28,40-29,61	0,445
CC, cm	90,99	89,99-91,98	88,71	86,76-90,66	91,79	90,41-93,17	0,016
MA, %	30,44	29,48-31,40	29,39	29,68-32,10	30,76	28,81-32,71	0,462
Colesterol total, mg/dL	183,95	180,60-187,30	184,42	178,01-190,83	183,74	179,13-188,34	0,866
LDL-c, mg/dL	117,44	114,41-120,47	119,13	113,15-125,12	116,97	112,78-121,15	0,564
HDL-c, mg/dL	41,74	40,88-42,61	41,08	39,41-42,75	41,97	40,86-43,08	0,400
Colesterol não HDL, mg/dL	142,19	138,75-145,63	143,31	136,69-149,93	141,76	136,92-146,60	0,715
Triglicerídeos, mg/dL	123,81	118,00-129,62	120,97	111,28-130,66	123,98	116,19-131,76	0,637
Glicose, mg/dL	94,56	92,32-96,80	93,39	89,08-97,70	94,89	92,10-97,68	0,571
PAS, mmHg	122,58	121,58-123,57	119,69	117,87-121,52	123,64	122,31-124,97	0,001
PAD, mmHg	74,53	73,84-75,23	73,63	72,43-74,82	74,84	73,91-75,77	0,129

Os dados são expressos como médias e intervalos de confiança de 95% (IC 95%). A significância estatística foi definida como p < 0,05 pelo teste de Wald. CC: circunferência da cintura; HDL-c: colesterol da lipoproteína de alta densidade; IC 95%: intervalo de confiança de 95%; IMC: índice de massa corporal; LDL-c: colesterol da lipoproteína de baixa densidade; MA: massa adiposa; PAD: pressão arterial diastólica; PAS: pressão arterial sistólica; SHIP-Brazil: Vida e Saúde em Pomerode.

**Tabela 3:** – Fatores de risco cardiometabólico segundo a preservação cultural germânica na coorte SHIP-Brazil, 2014-2018

Fator de risco cardiometabólico	Total (n = 727), n	% ponderada	Não (n = 218), n	% ponderada	Sim (n = 509), n	% ponderada	Valor de p
Excesso de peso	479	68.44	144	69.17	335	67.37	0.694
CC elevada	455	59.34	118	49.78	337	62.02	0.014
MA elevada	677	90.71	197	87.79	480	92.06	0.157
Colesterol total elevado	305	40.57	87	39.60	218	41.36	0.709
LDL-c elevado	87	11.45	28	12.17	59	11.54	0.839
HDL-c baixo	474	64.54	154	70.57	320	60.94	0.036
Colesterol não HDL elevado	221	29.06	64	28.60	157	29.29	0.873
Triglicerídeos elevados	177	23.01	51	22.11	126	22.96	0.828
Pré-diabetes ou diabetes tipo 2	249	31.77	67	29.52	182	32.75	0.464
Diabetes tipo 2	95	12.21	26	10.93	69	12.77	0.547
HAS	268	27.78	55	19.48	213	31.90	0.001
Risco intermediário ou alto de DCV aterosclerótica em 10 anos	181	23.51	34	12.23	147	25.60	< 0.001
Aterosclerose subclínica	74	7.31	7	2.15	67	8.46	0.001

Os dados são apresentados como frequências absolutas (n) e porcentagens ponderadas. As porcentagens foram calculadas utilizando pesos do plano amostral complexo e, portanto, podem diferir das proporções calculadas diretamente a partir das frequências observadas. A significância estatística foi definida como p < 0,05 pelo teste de Wald. CC: circunferência da cintura; DCV: doença cardiovascular; HAS: hipertensão arterial sistêmica; HDL-c: colesterol da lipoproteína de alta densidade; LDL-c: colesterol da lipoproteína de baixa densidade; MA: massa adiposa; SHIP-Brazil: Vida e Saúde em Pomerode.

A distribuição conjunta da PA segundo idade, sexo e preservação cultural germânica mostrou um padrão geral de maior PAS associado a essas características (Figura 2). Padrões semelhantes foram observados para o risco estimado de DCV e a aterosclerose subclínica (Figura 3).

**Figura 2:**
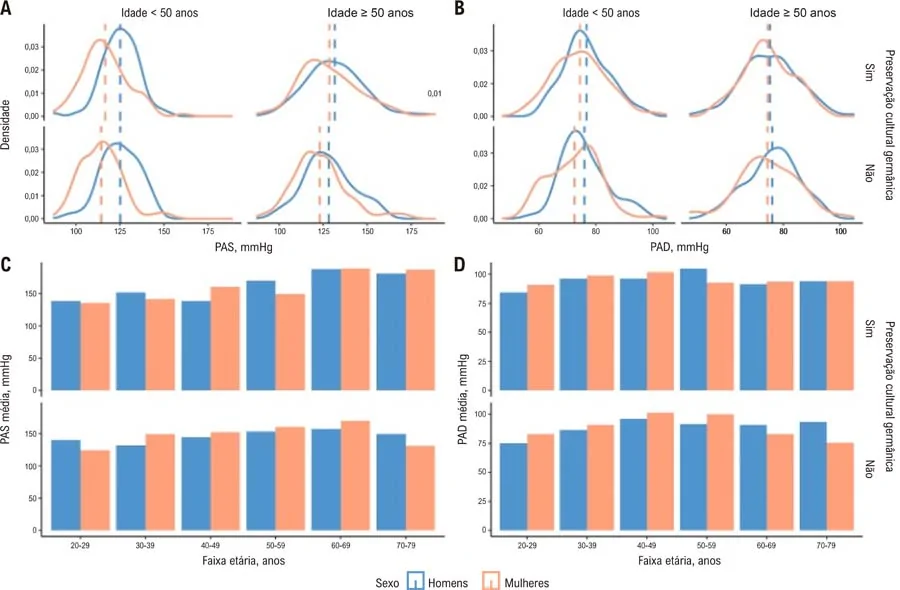
– Distribuições e valores médios da PAS e da PAD segundo a faixa etária, o sexo e a preservação cultural germânica. PAD: pressão arterial diastólica; PAS: pressão arterial sistólica.

**Figura 3:**
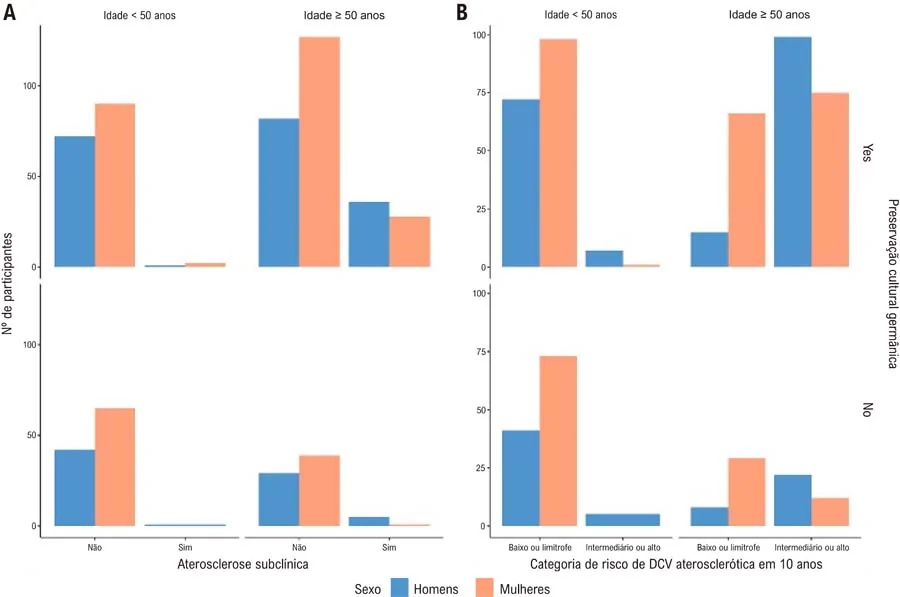
– Distribuição das categorias de risco de DCV aterosclerótica em 10 anos e da aterosclerose subclínica segundo a faixa etária, o sexo e a preservação cultural germânica. DCV: doença cardiovascular.

A Tabela 4 apresenta a distribuição dos estágios de HAS segundo a preservação cultural germânica, a idade e o sexo. A HAS foi mais frequente entre os participantes que preservavam a cultura germânica e aqueles com 50 anos ou mais. Embora os homens tenham apresentado maior prevalência geral de HAS, as mulheres apresentaram maiores frequências de HAS nos estágios 2 e 3, sugerindo diferenças relacionadas ao sexo na gravidade da HAS nessa população (todos p < 0,005).

**Tabela 4:** – Estágios de HAS segundo a preservação cultural germânica, a faixa etária e o sexo na coorte SHIP-Brazil, 2014-2018

Categoria de PA	Sem preservação cultural (n = 218), n (%)	Com preservação cultural (n = 509), n (%)	Valor de p	Idade < 50 anos (n = 327), n (%)	Idade ≥ 50 anos (n = 400), n (%)	Valor de p	Homens (n = 313), n (%)	Mulheres (n = 414), n (%)	Valor de p
Normal	153 (77,69)	288 (66,85)	0,014	238 (76,94)	203 (56,53)	< 0,001	161 (61,36)	280 (79,69)	0,001
Pré-hipertensão	31 (13,03)	91 (19,07)		51 (15,93)	71 (19,25)		73 (24,60)	49 (9,53)	
HAS estágio 1	23 (8,39)	78 (12,30)		23 (6,74)	78 (20,34)		56 (13,23)	45 (8,69)	
HAS estágios 2 ou 3	3 (0,89)	15 (1,78)		2 (0,39)	16 (3,88)		5 (0,81)	13 (2,09)	

Os dados são apresentados como frequências absolutas (n) e porcentagens ponderadas. As porcentagens foram calculadas utilizando pesos do plano amostral complexo e, portanto, podem diferir das proporções calculadas diretamente a partir das frequências observadas. Os pontos de corte da PA sistólica e diastólica foram definidos de acordo com as Diretrizes Brasileiras de Hipertensão Arterial – 2020.^35^ A significância estatística foi definida como p < 0,05 pelo teste de Wald. HAS: hipertensão arterial sistêmica; PA: pressão arterial; SHIP-Brazil: Vida e Saúde em Pomerode.

Embora os modelos de regressão linear múltipla não tenham demonstrado associações significativas entre a preservação cultural germânica e os marcadores contínuos de risco cardiometabólico (Material suplementar - Tabela S1), foram observadas associações significativas quando essas variáveis foram categorizadas por meio de pontos de corte clínicos estabelecidos.

Nos modelos de regressão logística multivariável estratificados por idade, a preservação cultural germânica esteve associada a chances aproximadamente 3 vezes maiores de HAS entre os participantes com 50 anos ou mais (*odds ratio* [OR]: 2,79; IC 95%: 1,49-5,22), após ajuste para sexo, raça e etnia, religião, nível socioeconômico e atividade física. Na mesma faixa etária, a preservação cultural germânica também esteve associada a chances aproximadamente 3 vezes maiores de aterosclerose subclínica com base na carga de placas carotídeas (OR: 3,34; IC 95%: 1,17-9,49) (Figura 4 e Figura Central). Não foram observadas associações consistentes entre os participantes com menos de 50 anos, conforme indicado pelos IC amplos e pela ausência de significância estatística (Figura 4 e Figura Central).

**Figura 4:**
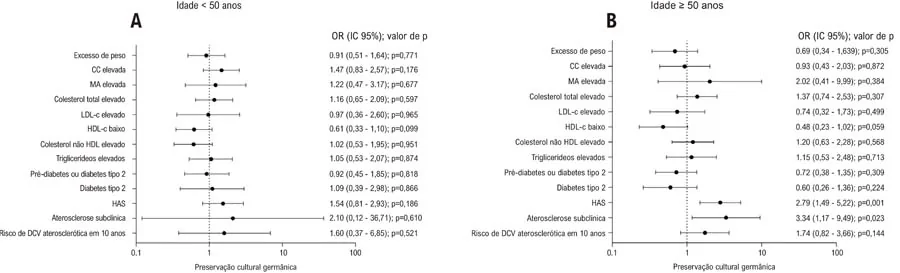
– Associação da preservação cultural germânica com fatores de risco cardiometabólico segundo a faixa etária. A preservação cultural germânica foi considerada a variável independente, e a presença de cada fator de risco cardiometabólico foi avaliada como variável dependente. Os resultados são apresentados como OR ajustadas e respectivos ICs 95%. A significância estatística foi estabelecida em p < 0,05. Os modelos de regressão logística multivariável foram ajustados para sexo, raça e etnia, atividade física, religião, nível socioeconômico e uso de medicamentos anti-hipertensivos. O uso de medicamentos anti-hipertensivos foi excluído do modelo de HAS por estar incluído na definição operacional desse desfecho. O modelo do risco de DCV aterosclerótica em 10 anos, estimado por meio das Equações de Coortes Agrupadas da ACC/AHA, foi ajustado apenas para atividade física, religião e nível socioeconômico, pois as demais covariáveis eram componentes das equações de risco. ACC: American College of Cardiology; AHA: American Heart Association; CC: circunferência da cintura; DCV: doença cardiovascular; HAS: hipertensão arterial sistêmica; HDL-c: colesterol da lipoproteína de alta densidade; LDL-c: colesterol da lipoproteína de baixa densidade; MA: massa adiposa; OR: odds ratio.

Foram realizadas análises adicionais para investigar mais detalhadamente a relação entre HAS e aterosclerose subclínica. Entre os participantes com 50 anos ou mais, a HAS esteve associada a chances quase 3 vezes maiores de aterosclerose subclínica (OR: 2,78; IC 95%: 1,33-5,81). Não foi observada associação estatisticamente significativa entre os participantes com menos de 50 anos (OR: 5,26; IC 95%: 0,70-39,70), e o IC amplo indicou precisão limitada nesse subgrupo.

Entre os participantes com 50 anos ou mais, a preservação cultural germânica permaneceu associada a maiores chances de HAS após ajuste adicional para aterosclerose subclínica (OR: 3,16; IC 95%: 1,08-9,27). Por outro lado, quando a aterosclerose subclínica foi considerada a variável dependente, a associação com a preservação cultural germânica foi atenuada após o ajuste para HAS (OR: 2,44; IC 95%: 0,87-6,86), sugerindo que a HAS pode explicar parcialmente a associação observada entre a preservação cultural germânica e a aterosclerose subclínica entre os participantes mais velhos.

Os participantes incluídos na amostra analítica foram semelhantes aos não incluídos quanto à distribuição por sexo, à faixa etária e à prevalência de HAS. Entretanto, a preservação cultural germânica foi mais frequente entre os participantes incluídos do que entre os não incluídos (66,15% vs 60,64%; p = 0,026). Para avaliar adicionalmente a robustez dos achados, foi realizada uma análise de sensibilidade utilizando a amostra mais ampla da linha de base do SHIP-Brazil com dados disponíveis sobre preservação cultural germânica e HAS (n = 2.447). Entre os participantes com 50 anos ou mais, a preservação cultural germânica permaneceu associada a maiores chances de HAS (OR: 1,51; IC 95%: 1,12-2,03; p = 0,007), corroborando a consistência dos achados principais (dados não apresentados).

## Discussão

Este estudo descreve o perfil de risco cardiovascular dos participantes do SHIP-Brazil e sua associação com a preservação cultural germânica. Entre os participantes com 50 anos ou mais, a preservação cultural germânica esteve associada a maiores chances de HAS e de aterosclerose subclínica com base na carga de placas carotídeas. Análises adicionais demonstraram que a HAS e a aterosclerose subclínica estavam estreitamente associadas nos adultos mais velhos, sugerindo que ambas podem coexistir como componentes de um perfil compartilhado de risco cardiovascular nessa população (Figura Central).

As comparações exploratórias com estudos de base populacional realizados na Alemanha devem ser interpretadas com cautela devido às diferenças de delineamento, composição demográfica, contexto socioeconômico e perfis de risco cardiovascular. Ainda assim, o SHIP-Brazil adotou métodos comparáveis aos utilizados nas coortes originais do SHIP, conduzidas no nordeste da Alemanha, possibilitando comparações contextuais entre populações com antecedentes históricos e migratórios compartilhados.

Dados do Bundes-Gesundheitssurvey 1998 (1997-1999; variação da PAS: 124-141 mmHg)^38^ e do German Health Interview and Examination Survey for Adults (2008-2011; variação da PAS: 124-130 mmHg)^19^ demonstraram redução da prevalência de HAS acompanhada por melhora do controle pressórico na Alemanha, apesar do aumento da obesidade.^22^ Em âmbito regional, as comparações entre o SHIP-START (variação da PAS: 128-148 mmHg) e o SHIP-TREND (variação da PAS: 124-132 mmHg), ambos realizados com indivíduos da Pomerânia Ocidental, no nordeste da Alemanha, demonstraram redução significativa dos valores médios de PA entre adultos com idade de 55-74 anos ao longo de aproximadamente 10 anos. A redução da prevalência de HAS observada em âmbito nacional na Alemanha também foi identificada na coorte mais recente do SHIP-TREND.^20^

Embora as comparações diretas entre essas populações sejam limitadas, o SHIP-Brazil incluiu descendentes de imigrantes provenientes da mesma região onde o SHIP-START e o SHIP-TREND foram conduzidos, e os estudos utilizaram procedimentos comparáveis para a aferição da PA. Na amostra total do SHIP-Brazil, a PAS média foi de 122 mmHg (variação: 87-189 mmHg), e a prevalência de HAS foi de 31,42%. Entre os participantes que preservavam a cultura germânica, a PAS média foi de 123 mmHg (variação: 87-189 mmHg), e a prevalência de HAS foi de 33,81%. A PAS média foi significativamente menor entre os participantes que não preservavam a cultura germânica (119 mmHg; variação: 91-170 mmHg), entre os quais a prevalência de HAS foi de 20,80%. A prevalência geral no SHIP-Brazil foi superior à prevalência de 24,5% relatada para a população adulta brasileira com base em dados coletados entre 2006 e 2019 pelo Sistema de Vigilância de Fatores de Risco e Proteção para Doenças Crônicas por Inquérito Telefônico, abrangendo aproximadamente 700.000 participantes.^15^ Essa diferença pode refletir, em parte, a distribuição etária da amostra do SHIP-Brazil.

A associação da idade com níveis elevados de PA e HAS está bem estabelecida. Caldeira et al.^15^ observaram redução anual de 0,38% na prevalência de HAS entre adultos brasileiros mais jovens, de 18,9% para 13,8%. Em contrapartida, a prevalência aumentou significativamente entre adultos com mais de 45 anos, passando de 35,2% em 2015 para 42,4% em 2019. Em consonância com essas tendências, a PAS e a prevalência de HAS permaneceram elevadas entre os participantes mais velhos do SHIP-Brazil que preservavam a cultura germânica.

A prevalência de HAS padronizada por idade entre migrantes residentes em Amsterdã foi elevada em diversos grupos populacionais, incluindo migrantes ganeses, surinameses de ascendência africana, surinameses de ascendência sul-asiática, indonésios e turcos.^4^ A HAS entre populações migrantes pode ser influenciada por etnia, alimentação, condições socioeconômicas e outros fatores contextuais. Neste estudo, práticas de estilo de vida associadas à cultura germânica podem ter contribuído para a maior prevalência de HAS. A preservação cultural germânica foi operacionalizada com base no uso relatado do idioma alemão no ambiente doméstico e na participação em associações comunitárias ou culturais germânicas que preservam costumes tradicionais, incluindo vestimentas, músicas e alimentos.^26^ A aculturação transcende o idioma e pode moldar as relações sociais, o sentimento de pertencimento e a identidade.^1^ A persistência da cultura germânica no Brasil reflete um processo contínuo de adaptação cultural e formação identitária ao longo das gerações,^39^ que pode ser relevante para o risco de HAS.^34^

Um aspecto metodológico importante é que as associações foram mais evidentes para os desfechos categorizados clinicamente, especialmente a HAS, do que para as medidas cardiometabólicas contínuas. Ainda assim, algumas medidas contínuas, incluindo a PAS e a circunferência da cintura, também diferiram de acordo com a preservação cultural germânica. Os achados mais robustos para os desfechos categóricos podem refletir, em parte, o uso de pontos de corte clínicos estabelecidos para identificar indivíduos com maior risco cardiovascular. Como essas categorias se basearam em critérios clínicos amplamente aceitos, elas aumentam a interpretabilidade clínica e epidemiológica dos achados. Entretanto, a categorização também pode resultar em perda de informação e criar diferenças aparentes em torno de limiares arbitrários; portanto, os achados das análises contínuas e categóricas devem ser interpretados em conjunto.

Estudos anteriores associaram com frequência a migração e a aculturação a perfis desfavoráveis de saúde cardiovascular. Entretanto, a imigração alemã para Pomerode teve início no século XIX, e a presente amostra foi composta predominantemente por descendentes, e não por imigrantes. Esse contexto histórico ressalta a persistência das práticas culturais ao longo de várias gerações. Haas et al.^40^ examinaram a prevalência de múltiplos fatores de risco para DCV entre indivíduos que migraram para a Alemanha e constataram que 62% dos migrantes provenientes da antiga União Soviética apresentavam três ou mais fatores de risco para DCV, proporção superior à observada entre os participantes que se autodeclararam alemães. Embora as populações e os contextos migratórios sejam diferentes, os participantes do SHIP-Brazil também apresentaram uma carga considerável de fatores de risco cardiovascular.

A obesidade e a circunferência da cintura elevada foram frequentes entre os participantes do SHIP-Brazil que preservavam a cultura germânica, com prevalências de 39,77% e 62,02%, respectivamente. A prevalência de obesidade observada foi aproximadamente 1,6 vez maior do que a relatada no Brasil (25,5%; Instituto Brasileiro de Geografia e Estatística, 2020), 1,7 vez maior do que a relatada na Alemanha (22,8%),^21^ 1,6 vez maior do que a observada no SHIP-START (24,7%) e 1,2 vez maior do que a observada no SHIP-TREND (32,0%).^41^ Esses achados indicam uma carga considerável de risco cardiometabólico entre os participantes do SHIP-Brazil que preservavam a cultura germânica. A preservação cultural germânica também esteve associada a chances aproximadamente 3 vezes maiores de aterosclerose subclínica entre os participantes com 50 anos ou mais. Esse achado é relevante porque o comportamento sedentário foi menos prevalente nesse grupo, enquanto outros fatores de risco comportamentais tradicionais, incluindo tabagismo e consumo de álcool, não apresentaram diferenças significativas. Entretanto, a associação com a aterosclerose subclínica foi atenuada e deixou de ser estatisticamente significativa após ajuste adicional para HAS, sugerindo que a HAS pode explicar parcialmente a associação observada.

A HAS pode promover disfunção endotelial e aterosclerose por meio de diversos mecanismos biológicos.^42,43^ A elevação sustentada da PA pode lesionar o endotélio vascular, reduzir a biodisponibilidade de óxido nítrico, aumentar a vasoconstrição,^44^ ativar vias pró-inflamatórias que envolvem a molécula de adesão celular vascular 1 e a molécula de adesão intercelular 1,^45^ além de facilitar a migração de monócitos, a oxidação do LDL-c e a formação de células espumosas.^46^ A HAS também pode induzir remodelamento vascular e rigidez arterial, perpetuando, assim, a lesão endotelial.^47^ Neste estudo, os participantes que preservavam a cultura germânica apresentaram elevada prevalência de HAS e eram mais velhos do que aqueles que não a preservavam. Tanto o envelhecimento quanto a HAS estão associados à rigidez arterial, à disfunção endotelial progressiva e ao comprometimento do reparo vascular, fatores que contribuem para o desenvolvimento da aterosclerose.^48^

A relação entre preservação cultural e risco cardiovascular é complexa. Embora este estudo tenha identificado uma associação da preservação cultural germânica com HAS e aterosclerose subclínica entre os participantes mais velhos, estudos com outras populações migrantes revelaram diferentes padrões de risco cardiovascular.^49^ Essa heterogeneidade ressalta a importância dos fatores ambientais e sociais na determinação do risco cardiovascular em populações com características culturais distintas. O contexto histórico pode ser particularmente relevante para o SHIP-Brazil, pois a maioria dos imigrantes que chegaram a Pomerode no século XIX era proveniente da Pomerânia, no nordeste da Alemanha. Assim, a “cultura germânica” examinada neste estudo pode refletir, mais especificamente, a cultura pomerana. Essas características ambientais, históricas e culturais podem contribuir para o perfil de risco cardiovascular observado em Pomerode. Embora a ancestralidade genética não tenha sido avaliada, não se pode excluir uma possível suscetibilidade genética associada à ancestralidade pomerana, o que justifica investigações adicionais. Em estudo anterior, constatamos que descendentes de primeira geração de imigrantes alemães apresentavam perfis de risco cardiovascular semelhantes aos dos imigrantes alemães, exceto por concentrações mais elevadas de HDL-c.^50^ No SHIP-Brazil, os fatores de risco cardiovascular também apresentaram associação significativa com placas carotídeas.^51^

### Limitações e pontos fortes do estudo

A migração e a aculturação envolvem processos múltiplos e complexos, dos quais apenas alguns foram examinados neste estudo. Portanto, algumas limitações devem ser reconhecidas. Primeiramente, devido ao delineamento transversal, não foi possível estabelecer relações temporais nem associações causais entre preservação cultural germânica, HAS e aterosclerose subclínica. Informações mais detalhadas sobre a história migratória familiar, a idade no momento da migração e o tempo de residência no Brasil, quando aplicável, bem como sobre a geração migratória, poderiam ter proporcionado uma melhor compreensão das associações entre preservação cultural e risco cardiovascular. Também não estavam disponíveis dados sobre ancestralidade genética. Embora a ancestralidade genética e a identidade cultural sejam construtos distintos, dados de ancestralidade poderiam ajudar a diferenciar suas respectivas contribuições para o perfil de risco cardiovascular observado nessa população.

Outra limitação diz respeito à estimativa do risco de DCV aterosclerótica em 10 anos por meio das Equações de Coortes Agrupadas da ACC/AHA. Embora essas equações tenham sido desenvolvidas e validadas em grandes coortes de base populacional e sejam amplamente utilizadas na pesquisa cardiovascular e na prática clínica, sua aplicabilidade a populações brasileiras com características raciais, étnicas e demográficas heterogêneas pode ser limitada. Além disso, as equações foram desenvolvidas para adultos com idade entre 40 e 79 anos, enquanto a amostra do SHIP-Brazil incluiu participantes com idade entre 20 e 79 anos. O uso dos valores permitidos mais próximos para participantes cujos dados estavam fora dos intervalos validados pode ter introduzido erro de mensuração e classificação incorreta do risco. Entretanto, ainda são escassas as ferramentas de predição de risco cardiovascular especificamente desenvolvidas e validadas para a população brasileira, o que respalda o uso cauteloso de equações estabelecidas internacionalmente em estudos epidemiológicos.

A preservação cultural germânica foi mais frequente entre os participantes incluídos na amostra analítica do que entre os não incluídos (66,15% vs 60,64%; p = 0,026), embora os grupos fossem semelhantes quanto à distribuição por sexo, à faixa etária e à prevalência de HAS. Essa diferença indica um possível viés de seleção relacionado à disponibilidade de dados completos e deve ser considerada na interpretação dos achados. Consequentemente, recomenda-se cautela ao generalizar os resultados para a população mais ampla da linha de base do SHIP-Brazil. Contudo, a análise de sensibilidade realizada na amostra ampliada do SHIP-Brazil demonstrou uma associação consistente entre a preservação cultural germânica e a HAS entre os participantes com 50 anos ou mais, corroborando a robustez dos achados principais.

Por fim, a classificação dicotômica da preservação cultural germânica pode não captar plenamente a heterogeneidade e a natureza multidimensional da identidade e das práticas culturais entre gerações e contextos sociais. Diferentes dimensões da preservação cultural, incluindo idioma, hábitos alimentares, tradições, redes sociais e participação em associações culturais, podem apresentar associações distintas com o risco cardiovascular. Essas dimensões devem ser avaliadas separadamente em estudos futuros.

Entre os pontos fortes deste estudo estão o uso de dados provenientes de uma coorte de base populacional de adultos com idade entre 20 e 79 anos residentes em Pomerode, município conhecido pela preservação da cultura germânica no Brasil. As análises consideraram o delineamento amostral complexo e incorporaram a ponderação pelo inverso da probabilidade para melhorar a representatividade populacional. A avaliação abrangente de múltiplos fatores cardiometabólicos e do risco estimado de DCV aterosclerótica em 10 anos permitiu uma caracterização mais integrada dos perfis de risco cardiovascular dos participantes. Além disso, a avaliação objetiva da carga de placas carotídeas reduziu a dependência de desfechos autorrelatados e forneceu uma medida direta de aterosclerose subclínica.

## Conclusão

Entre adultos com 50 anos ou mais residentes no Sul do Brasil, a preservação cultural germânica esteve associada a maiores chances de HAS e de aterosclerose subclínica. Entretanto, a associação com a aterosclerose subclínica foi atenuada após o ajuste para HAS, sugerindo que a HAS pode explicar parcialmente esse achado. Ao integrar indicadores socioculturais a medidas clínicas e vasculares, este estudo ressalta a importância de considerar o contexto cultural na caracterização do risco cardiovascular em populações com uma herança cultural de longa data.

## *Material suplementar

Para informação adicional, por favor, clique aqui. https://abccardiol.org/supplementary-material/2026/12310/2026-0095_Supplementary.pdf
