# Supplementary material for: Associação da Preservação Cultural Germânica Com o Risco Cardiovascular e a Aterosclerose Subclínica em Adultos do Sul do Brasil
Source: Arq Bras Cardiol. 2026 Nov 4;123(9):e20260095. [Article in Portuguese] doi: 10.36660/abc.20260095 (PMC13754395; doi:10.36660/abc.20260095)
Supplement: Supplementary file 1 [file 0066-782x-abc-123-9-e20260095-suppl01-en.pdf]

Supplementary Material

Table S1. Multivariate association between preservation of Germanic culture (independent variable) and cardiometabolic risk (dependent variable), according to age group.

**Cardiometabolic risk**	**Preservation of Germanic culture**					
	**<50 years**			**≥50 years**		
	**β**	**95% CI**	**p**	**β**	**95% CI**	**p**
Weight, kg	4.29	-0.07 - 8.65	0.054	-0.19	-4.79 - 4.41	0.934
BMI, kg/m²	0.34	-1.02 - 1.71	0.878	-0.02	-1.53 - 1.50	0.982
WC, cm	1.86	-1.11 - 4.83	0.219	0.91	-2.36 - 4.18	0.586
FM, %	-0.55	-3.97 - 2.86	0.748	0.31	-2.86 - 3.50	0.843
Total cholesterol, mg/dL	0.78	-11.08 - 12.65	0.896	1.24	-10.46 – 12.95	0.835
LDL-c, mg/dL	-0.94	-11.85 - 9.96	0.864	-2.04	-12.96 - 8.87	0.713
HDL-c, mg/dL	1.13	-1.40 - 3.68	0.379	2.48	-0.52 - 5.48	0.105
Non-HDL-c, mg/dL	-0.35	-12.54 - 11.84	0.955	-1.06	-12.85 - 10.72	0.859
Triglycerides, mg/dL	2.97	-13.77 – 19.72	0.727	4.06	-14.85 - 22.98	0.673
Glucose, mg/dL	1.59	-3.38 - 6.57	0.529	-6.61	-14.87 – 1.64	0.116
SBP, mmHg	2.00	-1.08 - 5.09	0.203	1.71	-2.29 - 5.72	0.401
DBP, mmHg	1.52	-0.59 - 3.65	0.159	-1.15	-3.80 – 1.49	0.391

β, beta coefficient; 95% CI, 95% confidence intervals; BMI, body mass index; WC, waist circumference; %FM, fat mass in percentage; LDL-c, low-density lipoprotein cholesterol; HDL-c, high-density lipoprotein cholesterol; TG, triglycerides; SBP, systolic blood pressure; DBP, diastolic blood pressure; p, value with statistical significance of 5% ($p<0.05$). Multiple linear regression models were adjusted for sex, race/ethnicity, religion, socioeconomic status, physical activity and use of antihypertensive medications.
