# Supplementary material for: Associação da Preservação Cultural Germânica Com o Risco Cardiovascular e a Aterosclerose Subclínica em Adultos do Sul do Brasil
Source: Arq Bras Cardiol. 2026 Nov 4;123(9):e20260095. [Article in Portuguese] doi: 10.36660/abc.20260095 (PMC13754395; doi:10.36660/abc.20260095)
Supplement: Translation

# Translation: Association of Germanic Cultural Preservation with Cardiovascular Risk and Subclinical Atherosclerosis in Adults from Southern Brazil

**DOI:** 10.36660/abc.20260095i

**Authors:** Ribanna Aparecida Marques Braga, Marcello Ricardo Paulista Markus, Marcus Dörr, Henry Völzke, Martin Bahls, Siegmar Starke, Sergio Luiz Zimmermann, Ernani Tiaraju de Santa Helena, Raul D. Santos, Luiz Aparecido Bortolotto, Joany Coronado, Nagila Raquel Teixeira Damasceno

**Affiliations:** 1 Brazil Instituto do Coração do Hospital das Clínicas da Faculdade de Medicina da Universidade de São Paulo, São Paulo, SP – Brazil; 2 Germany University Medicine Greifswald Department and Polyclinic of Internal Medicine B - Medicine Greifswald, Mecklenburg-Vorpommern – Germany; 3 Germany University Medicine Greifswald Institute of Community Medicine - Community Medicine Greifswald, Mecklenburg-Vorpommern – Germany; 4 Brazil Fundação Universidade Regional de Blumenau, Blumenau, SC – Brazil

**Keywords:** Heart Disease Risk Factors, Hypertension, Atherosclerosis

## Abstract

**Background:**

Migration involves cultural and lifestyle transitions that may influence cardiovascular disease (CVD) risk. Cultural preservation and adaptation may affect cardiovascular health through behavioral and social mechanisms.

**Objective:**

To assess the association of Germanic cultural preservation with CVD risk and subclinical atherosclerosis among residents of Pomerode, southern Brazil, stratified by age group and sex.

**Methods:**

This cross-sectional study used baseline data collected from 2014 to 2018 from the Study of Health in Pomerode cohort and included 727 individuals aged 20-79 years with complete data. Germanic cultural preservation was defined as regularly speaking German at home and participating in German cultural associations. Subclinical atherosclerosis was defined as the presence of three or more carotid plaques. 10-year atherosclerotic CVD risk was estimated using the American College of Cardiology/American Heart Association Pooled Cohort Equations. Statistical significance was set at p < 0.05.

**Results:**

Mean age was 43.7 years, and 51.9% of participants were women. Germanic cultural preservation was reported by 65.61% of participants and was more common among older adults, White individuals, Protestants, and individuals of middle socioeconomic status (p < 0.05). Participants reporting Germanic cultural preservation also had less sedentary behavior, greater use of antihypertensive medications, and a less favorable cardiometabolic profile. In adjusted age-stratified models, Germanic cultural preservation was associated with hypertension (odds ratio [OR]: 2.79; 95% CI: 1.49-5.22) and subclinical atherosclerosis (OR: 3.01; 95% CI: 1.07-8.40) among individuals aged 50 years or older. After adjustment for hypertension, the association with subclinical atherosclerosis was attenuated and was no longer statistically significant.

**Conclusions:**

Germanic cultural preservation was associated with hypertension and subclinical atherosclerosis among adults aged 50 years or older, highlighting the importance of considering cultural context when characterizing cardiovascular risk in aging populations.

## Introduction

Migration is a longstanding human phenomenon involving cultural, social, economic, and emotional changes that may influence health outcomes.^1^ Whether occurring within or across countries, migration may entail varying degrees of acculturation, characterized by changes in everyday practices, values, and identity as well as the preservation of cultural traditions and behaviors across generations.^2^ These cultural processes may influence health indicators and cardiovascular risk profiles.^3^

Differences in disease incidence, prevalence, and mortality between migrant and host populations are well documented.^4,5^ These differences vary according to the destination country, migrants’ region of origin and migration history, and the cardiovascular disease (CVD) outcome assessed.^6^ A systematic review identified heterogeneous patterns of CVD risk across Western Europe and North America; for example, North African migrants had a lower risk of stroke than the host population in the Netherlands.^5^ CVD risk profiles also differ between first- and second-generation migrants and host populations.^3^ Despite this heterogeneity, higher prevalences of obesity, type 2 diabetes,^8^ and hypertension have consistently been reported among migrant populations.^9^

Hypertension, in particular, remains the most prevalent cardiovascular risk factor worldwide^10,11^ and has consistently been associated with unfavorable cardiometabolic profiles among migrant populations.^10-12^ In Brazil, hypertension remains the leading cardiovascular risk factor, affecting approximately 30% of population.^13,14^ However, data on cardiovascular risk among migrant populations and their descendants in Brazil remain limited. Previous Brazilian studies involving migrant populations, including Japanese Brazilians and African migrants, have demonstrated a high burden of cardiometabolic risk.^15,16^ Similarly, studies conducted in Germany have shown that, despite the country’s robust health care system, hypertension and dyslipidemia remain underdiagnosed and undertreated, contributing to persistent cardiovascular risk.^17-21^ Nevertheless, the potential influence of Germanic cultural preservation on cardiovascular risk profiles and subclinical atherosclerosis in Brazil remains poorly understood.

Pomerode, located in southern Brazil, is known for its strong Germanic cultural heritage, with its language, traditions, and community practices preserved across generations. This setting provides a unique opportunity to examine the relationship between cultural preservation and cardiovascular health in a population with longstanding Germanic ancestry. Therefore, this study investigated the association of Germanic cultural preservation with cardiovascular risk profiles and subclinical atherosclerosis among adults from southern Brazil, with analyses stratified by age group and sex.

## Methods

### Study design and population

Current cross-sectional study used baseline data collected from 2014 to 2018 from Study of Health in Pomerode (SHIP-Brazil) cohort. Sampling strategy and methods used in this cohort were aligned with those of Study of Health in Pomerania (SHIP)-TREND cohort.^22^ Details of study design, conceptual framework, and procedures have been previously reported.^23^

A random sample was drawn across 12 strata defined by sex and age and included individuals aged 20-79 years. Sample size was calculated assuming an outcome prevalence of 50%, a precision of 5%, and a 95% confidence level, resulting in a required sample of 3,678 individuals. Individuals who had resided in the city of Pomerode, state of Santa Catarina, Brazil, for at least 6 months were eligible. Exclusion criteria were i) physical or cognitive impairment or mental illness that prevented individuals from completing the questionnaires or attending the Examination Center at University Hospital, Universidade Regional de Blumenau and ii) inability to speak Portuguese.

After losses and refusals of approximately 30% during baseline data collection, 2,488 individuals were interviewed. Analysis included a subsample of participants who provided biological samples and had complete data for the variables of interest. Of the participants interviewed, 753 provided blood and other biological samples. Subsequently, 26 participants without information on Germanic cultural preservation were excluded, resulting in a final analytical sample of 727 participants.

The study was conducted in accordance with the ethical principles of Declaration of Helsinki. All participants provided written informed consent. Study protocol was approved by research ethics committees under approval numbers 99559118.0.0000.5370 and 40160420.5.0000.5421.

### Sociodemographic and lifestyle data

Participants were interviewed using standardized procedures and structured questionnaires. Sociodemographic variables assessed included i) sex (men or women), ii) age (continuous and categorized as < 50 or ≥ 50 years), iii) self-reported race and ethnicity (White or non-White), iv) marital status (married, single, separated, or widowed), v) religion (Protestant, Catholic, no religious affiliation, or another religion), educational attainment (higher education [≥ 12 years], high school education [9-11 years], elementary education [5-8 years], or elementary education of < 4 years or no formal education), and socioeconomic status. Socioeconomic status was classified as high (class A), middle (class B), or low (classes C, D, and E) according to the Brazil Economic Classification Criteria.^24^

Lifestyle variables included i) risk of alcohol misuse, categorized as low or increased (moderate, high, or severe);^25^ ii) physical activity, assessed using the International Physical Activity Questionnaire and categorized as walking, moderate or vigorous physical activity, or sedentary behavior;^26^ and iii) smoking status, categorized as never, former, or current smoking. Data on use of antihypertensive medication were also collected.

### Germanic cultural preservation

Participants were classified as preserving Germanic culture if they reported both regularly speaking German at home and participating in German community or cultural associations that preserve German customs in Brazil, including traditional clothing, music, and food.^27^

### Anthropometric data

Weight and height were measured in kilograms and meters, respectively, and body mass index (BMI) was calculated as weight in kg divided by height in m^2^. Excess weight was defined as a BMI of 25 kg/m^2^ or greater in adults and 28 kg/m^2^ or greater in older adults, according to World Health Organization^28^ and Pan American Health Organization^29^ criteria, respectively.

Waist circumference was measured in centimeters and classified as high at 94 cm or greater for men and 80 cm or greater for women.^30^ Fat mass percentage was estimated using tetrapolar bioelectrical impedance analysis (BodyStat MDD1500) and classified as high at 25% or greater for men and 30% or greater for women.^31^

### Biochemistry analyses

Fasting blood samples were collected after a 10- to 12-hour fast in tubes containing ethylenediaminetetraacetic acid (1.0 mg/mL; BD, Brazil). Plasma total cholesterol, triglycerides, and high-density lipoprotein cholesterol (HDL-C) levels were measured using standard methods in an automated system. Low-density lipoprotein cholesterol (LDL-C) was calculated using the Friedewald equation^32^ when triglyceride levels were less than 400 mg/dL:


 LDL-C = (total cholesterol - HDL-C) - triglycerides/5

Lipid profile was classified according to Brazilian Guideline on Dyslipidemias and Prevention of Atherosclerosis – 2025^33^ as high total cholesterol (≥ 190 mg/dL), high LDL-C (≥ 160 mg/dL), low HDL-C (≤ 40 mg/dL for men and ≤ 50 mg/dL for women), high non-HDL-C (≥ 160 mg/dL), or high triglycerides (≥ 150 mg/dL).

Plasma glucose was measured using Glucose PAP Liquiform (Labtest Diagnóstica S.A., Lagoa Santa, MG, Brazil). Thresholds of 100 mg/dL or greater for prediabetes and 126 mg/dL or greater for type 2 diabetes were used.^34^ Blood pressure (BP) levels were classified according to Brazilian Guidelines of Hypertension – 2020.^35^ Systolic BP (SBP) and diastolic BP (DBP) were measured three times with a sphygmomanometer after participants had remained seated and at rest for 5 minutes. The mean of second and third measurements was used in analyses.

### Subclinical atherosclerosis

Extracranial carotid arteries were examined using B-mode ultrasonography with a broadband linear transducer operating at 13 MHz. Carotid plaque was defined as either i) a focal protrusion of intima-media complex into vessel lumen above the surrounding intima-media layer or ii) a focal increase in echogenicity in a longitudinal section accompanied by an irregular luminal surface, even in absence of substantial protrusion into the lumen.

Examinations were performed by trained and certified examiners, who assessed each arterial segment separately in longitudinal and cross-sectional views, with particular attention to identifying atherosclerotic plaques. Vessels were examined from multiple insonation angles, ranging from the most anterior to the most posterior positions, to improve the detection of small and eccentric plaques.

Common carotid, internal carotid, and external carotid arteries and carotid bifurcations were systematically examined bilaterally. Subclinical atherosclerosis was defined as the presence of three or more carotid plaques, reflecting carotid plaque burden.

### 10-year risk of CVD

The 10-year risk of hard atherosclerotic CVD, which is defined as nonfatal myocardial infarction, coronary heart disease death, or fatal or nonfatal stroke, was estimated using American College of Cardiology (ACC)/American Heart Association (AHA) Pooled Cohort Equations.^36^ These equations incorporate sex, age, race, SBP, total cholesterol, HDL-C, diabetes status, antihypertensive treatment, and smoking status. For input values outside the ranges accepted by the equations, the nearest allowable value was used. Estimated 10-year risk of atherosclerotic CVD was classified as low (< 5%), borderline (5% to < 7.5%), intermediate (7.5% to < 20%), or high (≥ 20%). For analyses, participants were subsequently grouped as having low or borderline risk versus intermediate or high risk.

### Statistical analysis

A post hoc statistical power analysis was performed using G*Power, version 3.1.9.7,^37^ for multiple linear regression. Parameters included a medium effect size (*f*^2^ = 0.15), an α level of 0.05, a sample size of 727, and four predictors. A two-sided test yielded a statistical power of 1.00, indicating adequate power to detect a medium effect. Analysis had 722 degrees of freedom and a critical *t* value of 1.96.

Because age and sex distributions of SHIP-Brazil analytical sample differed from those of population of Pomerode, a two-step inverse probability weighting approach was used to minimize potential selection bias and improve population representativeness. First, each participant’s probability of inclusion in analytical sample was estimated according to demographic characteristics, including age and sex. Second, inverse probability weights were calculated as the inverse of estimated selection probabilities and incorporated into all analyses. This approach assigned proportionally greater weight to individuals from groups that were underrepresented in the analytical sample, thereby improving comparability between the analytical sample and the population of Pomerode.

Descriptive analyses were performed, with continuous variables reported as means and 95% CIs and categorical variables as numbers and percentages. Wald tests were used to evaluate associations of Germanic cultural preservation with cardiovascular risk factors and subclinical atherosclerosis based on carotid plaque burden. Multivariable regression models were subsequently fitted, with adjustment for covariates selected a priori according to their epidemiological relevance and associations identified in the bivariate analyses (p < 0.05). Sex was retained in all adjusted models because of its established relevance to cardiovascular risk.

Multiple linear regression was used to evaluate association of Germanic cultural preservation with individual cardiometabolic risk factors, stratified by age group and adjusted for sex, race and ethnicity, religion, socioeconomic status, physical activity, and use of antihypertensive medication. Before fitting models, the assumptions of linearity, normally distributed residuals, homoscedasticity, independence of errors, and absence of multicollinearity were evaluated. All assumptions required for multiple linear regression were met.

Multivariable binary logistic regression models were also fitted to assess the associations of Germanic cultural preservation with cardiovascular risk markers and subclinical atherosclerosis, stratified by age group. Covariates were selected according to theoretical and statistical criteria to minimize collinearity and avoid overadjustment. The hypertension model was adjusted for sex, race and ethnicity, religion, socioeconomic status, and physical activity because use of antihypertensive medication was already included in the operational definition of hypertension. For the estimated 10-year risk of atherosclerotic CVD, physical activity, religion, and socioeconomic status were included as covariates because the remaining variables were components of the risk equations. Adjustment set used in the multiple linear regression models was retained for other outcomes.

All statistical analyses were performed using Stata/SE, version 17 (StataCorp LLC); GraphPad Prism, version 8.0.1 (GraphPad Software); or R, version 4.4.1 (R Foundation for Statistical Computing), with the “table1” and “ggplot2” packages. Statistical significance was defined as a two-sided p < 0.05

## Results

Analytical sample comprised 727 participants (Figure 1), of whom 51.92% were women. Mean age was 43.74 (95% CI: 43.51-43.96) years. Germanic cultural preservation was identified in 65.61% of participants (unweighted n = 509) and was more common among older participants and those who self-identified as White. Overall, 61.36% (unweighted n = 486) reported speaking German at home, and 21.24% (unweighted n = 176) reported participating in German community or cultural associations. Among participants classified as preserving Germanic culture, 93.53% spoke German at home and 32.38% participated in such associations. These participants were also more likely to be Protestant, have middle socioeconomic status, report less sedentary behavior, and use antihypertensive medications (Table 1).

**Figure 1:** – Flowchart of study sample selection and exclusion criteria.)

**Table 1:** – Sociodemographic and lifestyle characteristics according to Germanic cultural preservation

Characteristic	Total, n	Total, weighted %	No, n	No, weighted %	Yes, n	Yes, weighted %	p-value
**Sex**							0.531
Men	313	48.07	89	46.26	224	48.99	
Women	414	51.92	129	53.73	285	51.00	
**Age group, years**							< 0.001
< 50	327	65.42	133	77.26	194	59.37	
≥ 50	400	34.57	85	22.73	315	40.62	
**Race and ethnicity**							< 0.001
Non-White	41	5.66	34	16.71	7	2.45	
White	684	94.34	184	83.29	500	97.55	
**Marital status**							0.251
Married	549	71.44	167	72.92	382	70.66	
Single	83	20.51	27	20.09	56	20.73	
Separated	30	3.53	14	4.50	16	3.02	
Widowed	65	4.51	10	2.47	55	5.57	
**Religion**							0.014
Protestant	498	65.49	79	34.88	419	81.56	
Catholic	204	29.79	126	57.60	78	15.20	
No religious affiliation	12	2.26	7	3.38	5	1.67	
Other	12	2.46	6	4.14	6	1.58	
**Educational attainment, years**							0.062
Higher education (≥ 12)	87	18.95	32	18.47	55	19.20	
High school education (9-11)	161	33.25	57	34.52	104	32.58	
Elementary education (5-8)	145	18.94	69	29.54	76	13.35	
Elementary education (< 4) or no formal education	318	28.84	57	17.45	261	34.84	
**Socioeconomic status**							0.002
High	116	17.58	25	11.99	91	20.52	
Middle	454	63.72	139	63.60	315	63.79	
Low	156	18.68	54	24.40	102	15.67	
**Risk of alcohol misuse**							0.194
Low	529	74.30	164	70.37	365	64.14	
High	183	25.70	54	29.63	129	35.86	
**Physical activity**							0.047
Walking or moderate/vigorous activity	445	65.06	128	60.64	317	69.67	
Sedentary behavior	239	34.94	80	39.36	159	30.33	
**Smoking status**							0.391
Never smoker	499	69.60	149	71.24	350	75.83	
Former smoker	156	21.76	52	21.51	104	17.13	
Current smoker	62	8.65	17	7.25	45	7.04	
**Use of antihypertensive** medication							0.019
Yes	211	31.21	43	15.82	168	23.98	

Data are presented as absolute frequencies (n) and weighted percentages. Percentages were calculated using complex sampling weights and may therefore differ from proportions calculated directly from the observed frequencies. Statistical significance was defined as p < 0.05 using the Wald test.

As shown in Table 2, Germanic cultural preservation was associated with higher mean weight, waist circumference, and SBP (all p < 0.005). Consistent with these findings, hypertension was more prevalent among participants who preserved Germanic culture than among those who did not (33.81% vs 20.80%). Excess weight was highly prevalent in the overall sample (68.44%), and 37.59% of participants had obesity. The prevalence of obesity was 39.77% among participants who preserved Germanic culture (p = 0.177; data not shown). Participants who preserved Germanic culture also had a higher estimated CVD risk and a greater prevalence of subclinical atherosclerosis (Table 3).

**Table 2:** – Cardiometabolic characteristics according to Germanic cultural preservation in the SHIP-Brazil cohort, 2014-2018

Cardiometabolic characteristic	Total (n = 727), mean	95% CI	No (n = 218), mean	95% CI	Yes (n = 509), mean	95% CI	p-value
Weight, kg	81.07	79.64-82.51	78.58	75.68-81.48	82.69	80.73-84.65	0.027
BMI, kg/m^2^	28.91	28.45-29.37	28.59	27.73-29.45	29.01	28.40-29.61	0.445
WC, cm	90.99	89.99-91.98	88.71	86.76-90.66	91.79	90.41-93.17	0.016
FM, %	30.44	29.48-31.40	29.39	29.68-32.10	30.76	28.81-32.71	0.462
Total cholesterol, mg/dL	183.95	180.60-187.30	184.42	178.01-190.83	183.74	179.13-188.34	0.866
LDL-C, mg/dL	117.44	114.41-120.47	119.13	113.15-125.12	116.97	112.78-121.15	0.564
HDL-C, mg/dL	41.74	40.88-42.61	41.08	39.41-42.75	41.97	40.86-43.08	0.400
Non-HDL-C, mg/dL	142.19	138.75-145.63	143.31	136.69-149.93	141.76	136.92-146.60	0.715
Triglycerides, mg/dL	123.81	118.00-129.62	120.97	111.28-130.66	123.98	116.19-131.76	0.637
Glucose, mg/dL	94.56	92.32-96.80	93.39	89.08-97.70	94.89	92.10-97.68	0.571
SBP, mmHg	122.58	121.58-123.57	119.69	117.87-121.52	123.64	122.31-124.97	0.001
DBP, mmHg	74.53	73.84-75.23	73.63	72.43-74.82	74.84	73.91-75.77	0.129

Data are expressed as means and 95% CIs. Statistical significance was defined as p < 0.05 using the Wald test. BMI: body mass index; DBP: diastolic blood pressure; FM: fat mass; HDL-C: high-density lipoprotein cholesterol; LDL-C: low-density lipoprotein cholesterol; SBP: systolic blood pressure; SHIP-Brazil: Study of Health in Pomerode; WC: waist circumference.

**Table 3:** – Cardiometabolic risk factors according to Germanic cultural preservation in the SHIP-Brazil cohort, 2014-2018

Cardiometabolic risk factor	Total (n = 727), n	Weighted %	No (n = 218), n	Weighted %	Yes (n = 509), n	Weighted %	p-value
Excess weight	479	68.44	144	69.17	335	67.37	0.694
High WC	455	59.34	118	49.78	337	62.02	0.014
High FM	677	90.71	197	87.79	480	92.06	0.157
High total cholesterol	305	40.57	87	39.60	218	41.36	0.709
High LDL-C	87	11.45	28	12.17	59	11.54	0.839
Low HDL-C	474	64.54	154	70.57	320	60.94	0.036
High non-HDL-C	221	29.06	64	28.60	157	29.29	0.873
High triglycerides	177	23.01	51	22.11	126	22.96	0.828
Prediabetes or type 2 diabetes	249	31.77	67	29.52	182	32.75	0.464
Type 2 diabetes	95	12.21	26	10.93	69	12.77	0.547
Hypertension	268	27.78	55	19.48	213	31.90	0.001
Intermediate or high 10-year atherosclerotic CVD risk	181	23.51	34	12.23	147	25.60	< 0.001
Subclinical atherosclerosis	74	7.31	7	2.15	67	8.46	0.001

Data are presented as absolute frequencies (n) and weighted percentages. Percentages were calculated using complex sampling weights and may therefore differ from proportions calculated directly from observed frequencies. Statistical significance was defined as p < 0.05 using the Wald test. CVD: cardiovascular disease; FM: fat mass; HDL-C: high-density lipoprotein cholesterol; LDL-C: low-density lipoprotein cholesterol; SHIP-Brazil: Study of Health in Pomerode; WC: waist circumference.

Joint distribution of BP according to age, sex, and Germanic cultural preservation showed a general pattern of higher SBP across these characteristics (Figure 2). Similar patterns were observed for estimated CVD risk and subclinical atherosclerosis (Figure 3).

**Figure 2:** – Distributions and mean values of SBP and DBP according to age group, sex, and Germanic cultural preservation. DBP: diastolic blood pressure; SBP: systolic blood pressure.

**Figure 3:** – Distribution of 10-year atherosclerotic CVD risk categories and subclinical atherosclerosis according to age group, sex, and Germanic cultural preservation. CVD: cardiovascular disease.

Table 4 presents the distribution of hypertension stages according to Germanic cultural preservation, age, and sex. Hypertension was more frequent among participants who preserved Germanic culture and those aged 50 years or older. Although men had a higher overall prevalence of hypertension, women had higher frequencies of stage 2 and stage 3 hypertension, which suggests sex-specific differences in hypertension severity in this population (all p < 0.005).

**Table 4:** – Hypertension stages according to Germanic cultural preservation, age group, and sex in the SHIP-Brazil cohort, 2014-2018

BP category	No cultural preservation (n = 218), n (%)	Cultural preservation (n = 509), n (%)	p-value	Age < 50 years (n = 327), n (%)	Age ≥ 50 years (n = 400), n (%)	p-value	Men (n = 313), n (%)	Women (n = 414), n (%)	p-value
Normal	153 (77.69)	288 (66.85)	0.014	238 (76.94)	203 (56.53)	< 0.001	161 (61.36)	280 (79.69)	0.001
Prehypertension	31 (13.03)	91 (19.07)		51 (15.93)	71 (19.25)		73 (24.60)	49 (9.53)	
Stage 1 hypertension	23 (8.39)	78 (12.30)		23 (6.74)	78 (20.34)		56 (13.23)	45 (8.69)	
Stage 2 or 3 hypertension	3 (0.89)	15 (1.78)		2 (0.39)	16 (3.88)		5 (0.81)	13 (2.09)	

Data are presented as absolute frequencies (n) and weighted percentages. Percentages were calculated using complex sampling weights and may therefore differ from proportions calculated directly from observed frequencies. Systolic and diastolic BP cutoff values were defined according to Brazilian Guidelines of Hypertension – 2020.35 Statistical significance was defined as p < 0.05 using the Wald test. BP: blood pressure; SHIP-Brazil: Study of Health in Pomerode.

Although the multiple linear regression models showed no significant associations between Germanic cultural preservation and continuous cardiometabolic risk markers (Supplementary Material - Table S1), significant associations were observed when these variables were categorized using established clinical cutoff values.

In age-stratified multivariable logistic regression models, Germanic cultural preservation was associated with approximately 3-fold greater odds of hypertension among participants aged 50 years or older (odds ratio [OR]: 2.79; 95% CI: 1.49-5.22) after adjustment for sex, race and ethnicity, religion, socioeconomic status, and physical activity. In the same age group, Germanic cultural preservation was also associated with approximately 3-fold greater odds of subclinical atherosclerosis based on carotid plaque burden (OR: 3.34; 95% CI: 1.17-9.49) (Figure 4 and Central Illustration). No consistent associations were observed among participants younger than 50 years, as indicated by wide CIs and lack of statistical significance (Figure 4 and Central Illustration).

**Figure 4:** – Association of Germanic cultural preservation with cardiometabolic risk factors according to age group. Germanic cultural preservation was the independent variable, and the presence of individual cardiometabolic risk factors was assessed as dependent variable. Results are presented as adjusted ORs and 95% CIs. Statistical significance was set at p < 0.05. Multivariable logistic regression models were adjusted for sex, race and ethnicity, physical activity, religion, socioeconomic status, and use of antihypertensive medication. Use of antihypertensive medication was excluded from hypertension model because it was included in operational definition of hypertension. Model for 10-year atherosclerotic CVD risk estimated using the ACC/AHA Pooled Cohort Equations was adjusted only for physical activity , religion, and socioeconomic status because the remaining covariates were components of the risk equations. ACC: American College of Cardiology; AHA: American Heart Association; CVD: cardiovascular disease; FM: fat mass; HDL-C: high-density lipoprotein cholesterol; LDL-C: low-density lipoprotein cholesterol; OR: odds ratio; WC: waist circumference.

Additional analyses were conducted to further examine the relationship between hypertension and subclinical atherosclerosis. Among participants aged 50 years or older, hypertension was associated with nearly 3-fold greater odds of subclinical atherosclerosis (OR: 2.78; 95% CI: 1.33-5.81). No statistically significant association was observed among participants younger than 50 years (OR: 5.26; 95% CI: 0.70-39.70), and the wide CI indicated limited precision in this subgroup.

Among participants aged 50 years or older, Germanic cultural preservation remained associated with greater odds of hypertension after further adjustment for subclinical atherosclerosis (OR: 3.16; 95% CI: 1.08-9.27). Conversely, when subclinical atherosclerosis was the dependent variable, the association with Germanic cultural preservation was attenuated after adjustment for hypertension (OR: 2.44; 95% CI: 0.87-6.86), suggesting that hypertension may partly account for the observed association between Germanic cultural preservation and subclinical atherosclerosis among older participants.

Participants included in the analytical sample were similar to those who were not included with respect to sex distribution, age group, and hypertension prevalence. However, Germanic cultural preservation was more frequent among included participants than among those not included (66.15% vs 60.64%; p = 0.026). To further assess the robustness of the findings, a sensitivity analysis was performed using the broader SHIP-Brazil baseline sample with available data on Germanic cultural preservation and hypertension (n = 2,447). Among participants aged 50 years or older, Germanic cultural preservation remained associated with greater odds of hypertension (OR: 1.51; 95% CI: 1.12-2.03; p = 0.007), supporting the consistency of primary findings (data not shown).

## Discussion

Current study describes cardiovascular risk profile of participants in SHIP-Brazil and its association with Germanic cultural preservation. Among participants aged 50 years or older, Germanic cultural preservation was associated with greater odds of hypertension and subclinical atherosclerosis based on carotid plaque burden. Additional analyses showed that hypertension and subclinical atherosclerosis were closely associated in older adults, which suggests they may coexist as components of a shared cardiovascular risk profile in this population (Central Illustration).

Exploratory comparisons with population-based studies conducted in Germany should be interpreted cautiously because of differences in study design, demographic composition, socioeconomic context, and cardiovascular risk profiles. Nevertheless, SHIP-Brazil adopted methods comparable to those used in original SHIP cohorts from northeastern Germany, allowing contextual comparisons between populations with shared historical and migratory backgrounds.

Data from Bundes-Gesundheitssurvey 1998 (1997-1999; SBP range: 124-141 mmHg)^38^ and German Health Interview and Examination Survey for Adults (2008-2011; SBP range: 124-130 mmHg)^19^ showed a decrease in prevalence of hypertension accompanied by improvements in BP control in Germany, despite an increase in obesity.^22^ At regional level, comparisons between SHIP-START (SBP range: 128-148 mmHg) and SHIP-TREND (SBP range: 124-132 mmHg), both conducted among individuals from Western Pomerania in northeastern Germany, showed a significant decrease in mean BP among adults aged 55-74 years over approximately 10 years. Nationwide decrease in hypertension prevalence observed in Germany was also evident in the more recent SHIP-TREND cohort.^20^

Although direct comparisons between these populations are limited, SHIP-Brazil included descendants of immigrants from the same region in which SHIP-START and SHIP-TREND were conducted, and the studies used comparable procedures for measuring BP. In the overall SHIP-Brazil sample, mean SBP was 122 mmHg (range: 87-189 mmHg), and prevalence of hypertension was 31.42%. Among participants who preserved Germanic culture, mean SBP was 123 mmHg (range: 87-189 mmHg), and hypertension prevalence was 33.81%. Mean SBP was significantly lower among participants who did not preserve Germanic culture (119 mmHg; range: 91-170 mmHg), among whom prevalence of hypertension was 20.80%. The overall prevalence in SHIP-Brazil exceeded the 24.5% prevalence reported for the Brazilian adult population in Brazilian Surveillance System of Risk and Protective Factors for Chronic Diseases by Telephone Survey data collected from 2006 to 2019 (approximately 700,000 participants).^15^ This difference may partly reflect the age distribution of the SHIP-Brazil sample.

The association of age with increased BP and hypertension is well established. Caldeira et al.^15^ observed an annual decrease of 0.38% in prevalence of hypertension among younger Brazilian adults, from 18.9% to 13.8%. By contrast, prevalence increased significantly among adults older than 45 years, from 35.2% in 2015 to 42.4% in 2019. Consistent with these trends, SBP and prevalence of hypertension remained increased among older SHIP-Brazil participants who preserved Germanic culture.

Age-standardized prevalence of hypertension among migrants living in Amsterdam was high across several population groups, including Ghanaian, African Surinamese, South Asian Surinamese, Indonesian, and Turkish migrants.^4^ Hypertension among migrant populations may be influenced by ethnicity, diet, socioeconomic conditions, and other contextual factors. In current study, lifestyle practices associated with Germanic culture may have contributed to higher prevalence of hypertension. Germanic cultural preservation was operationalized using reported use of the German language at home and participation in German community or cultural associations that preserve traditional customs, including clothing, music, and food.^26^ Acculturation extends beyond language and may shape social relationships, emotional belonging, and identity.^1^ Persistence of Germanic culture in Brazil reflects an ongoing process of cultural adaptation and identity formation across generations,^39^ which may be relevant to hypertension risk.^34^

An important methodological consideration is that associations were more evident for clinically categorized outcomes, particularly hypertension, than for continuous cardiometabolic measures. Nevertheless, some continuous measures, including SBP and waist circumference, also differed according to Germanic cultural preservation. Stronger findings for categorical outcomes may partly reflect the use of clinically established cutoff values intended to identify individuals at increased cardiovascular risk. Because these categories were based on widely accepted clinical criteria, they enhance clinical and epidemiological interpretability of findings. However, categorization may also reduce information and create apparent differences around arbitrary thresholds; therefore, the findings from continuous and categorical analyses should be interpreted together.

Previous studies have frequently associated migration and acculturation with unfavorable cardiovascular health profiles. However, German immigration to Pomerode began in the 19th century, and the present sample primarily comprised descendants rather than immigrants. This historical context underscores the persistence of cultural practices across multiple generations. Haas et al.^40^ examined the prevalence of multiple CVD risk factors among individuals who migrated to Germany and found that 62% of migrants from former Soviet Union had three or more CVD risk factors, a higher proportion than that observed among participants who self-identified as German. Although the populations and migration contexts differ, SHIP-Brazil participants similarly exhibited a substantial burden of cardiovascular risk factors.

Obesity and high waist circumference were common among SHIP-Brazil participants who preserved Germanic culture, with prevalences of 39.77% and 62.02%, respectively. The observed prevalence of obesity was approximately 1.6 times that reported for Brazil (25.5%; Brazilian Institute of Geography and Statistics, 2020), 1.7 times that reported for Germany (22.8%),^21^ 1.6 times that observed in SHIP-START (24.7%), and 1.2 times that observed in SHIP-TREND (32.0%).^41^ These findings indicate a substantial burden of cardiometabolic risk among SHIP-Brazil participants who preserved Germanic culture. Germanic cultural preservation was also associated with approximately 3-fold greater odds of subclinical atherosclerosis among participants aged 50 years or older. This finding is noteworthy because sedentary behavior was less prevalent in this group, whereas other traditional behavioral risk factors, including smoking and alcohol consumption, did not differ significantly. However, the association with subclinical atherosclerosis was attenuated and was no longer statistically significant after additional adjustment for hypertension, which suggests hypertension may partly account for the observed association.

Hypertension may promote endothelial dysfunction and atherosclerosis through multiple biological mechanisms.^42,43^ Sustained increase of BP can damage the vascular endothelium, reduce nitric oxide bioavailability, increase vasoconstriction,^44^ activate proinflammatory pathways involving vascular cell adhesion molecule 1 and intercellular adhesion molecule 1,^45^ and facilitate monocyte migration, LDL-C oxidation, and foam cell formation.^46^ Hypertension can also induce vascular remodeling and arterial stiffness, thereby perpetuating endothelial injury.^47^ In current study, participants who preserved Germanic culture had a high prevalence of hypertension and were older than those who did not. Both aging and hypertension are associated with arterial stiffness, progressive endothelial dysfunction, and impaired vascular repair, all of which contribute to the development of atherosclerosis.^48^

The relationship between cultural preservation and cardiovascular risk is complex. While this study identified an association between Germanic cultural preservation and hypertension and subclinical atherosclerosis among older participants, studies of other migrant populations have revealed different patterns of cardiovascular risk.^49^ This heterogeneity highlights the importance of environmental and social factors in shaping cardiovascular risk in populations with distinct cultural characteristics. Historical context may be particularly relevant to SHIP-Brazil because most 19th-century immigrants to Pomerode came from Pomerania in northeastern Germany. Thus, the “Germanic culture” examined in this study may more specifically reflect Pomeranian culture. These environmental, historical, and cultural characteristics may contribute to the cardiovascular risk profile observed in Pomerode. Although genetic ancestry was not assessed, potential genetic susceptibility associated with Pomeranian ancestry cannot be excluded and warrants further investigation. In a previous study, we found that first-generation descendants of German immigrants had cardiovascular risk profiles similar to those of German immigrants, except for higher HDL-C levels.^50^ In SHIP-Brazil, cardiovascular risk factors were also significantly associated with carotid plaques.^51^

### Limitations and strengths of study

Migration and acculturation involve multiple, complex processes, only some of which were examined in this study. Several limitations should therefore be acknowledged. First, because of the cross-sectional design, temporal relationships and causal associations among Germanic cultural preservation, hypertension, and subclinical atherosclerosis cannot be established. More detailed information on family migration history, age at migration and duration of residence in Brazil when applicable, and generational status could have provided a better understanding of the associations between cultural preservation and cardiovascular risk. Genetic ancestry data were also unavailable. Although genetic ancestry and cultural identity are distinct constructs, ancestry data could help distinguish their respective contributions to the cardiovascular risk profile observed in this population.

Another limitation concerns the estimation of 10-year atherosclerotic CVD risk using the ACC/AHA Pooled Cohort Equations. Although these equations were developed and validated in large population-based cohorts and are widely used in cardiovascular research and clinical practice, their applicability to Brazilian populations with heterogeneous racial, ethnic, and demographic characteristics may be limited. Furthermore, the equations were developed for adults aged 40-79 years, whereas the SHIP-Brazil sample included participants aged 20-79 years. Using the nearest allowable input values for participants outside the validated ranges may have introduced measurement error and risk misclassification. Nevertheless, cardiovascular risk prediction tools specifically developed and validated for the Brazilian population remain limited, supporting the cautious use of internationally established equations in epidemiological studies.

Germanic cultural preservation was more frequent among participants included in the analytical sample than among those not included (66.15% vs 60.64%; p = 0.026), although the groups were similar with respect to sex distribution, age group, and hypertension prevalence. This difference indicates potential selection bias related to availability of complete data and should be considered when interpreting the findings. Consequently, caution is warranted when generalizing the results to the broader SHIP-Brazil baseline population. However, the sensitivity analysis conducted in the larger SHIP-Brazil sample showed a consistent association between Germanic cultural preservation and hypertension among participants aged 50 years or older, supporting the robustness of the primary findings.

Finally, dichotomous classification of Germanic cultural preservation may not fully capture the heterogeneity and multidimensional nature of cultural identity and practices across generations and social contexts. Different dimensions of cultural preservation, including language, dietary habits, traditions, social networks, and participation in cultural associations, may have distinct associations with cardiovascular risk. These dimensions should be assessed separately in future studies.

Strengths of current study include its use of data from a population-based cohort of adults aged 20-79 years residing in Pomerode, a city known for Germanic cultural preservation in Brazil. Analyses accounted for complex sampling design and incorporated inverse probability weighting to improve population representativeness. Comprehensive assessment of multiple cardiometabolic factors and estimated 10-year atherosclerotic CVD risk allowed a more integrated characterization of participants’ cardiovascular risk profiles. In addition, objective assessment of carotid plaque burden reduced reliance on self-reported outcomes and provided a direct measure of subclinical atherosclerosis.

## Conclusion

Among adults aged 50 years or older living in southern Brazil, Germanic cultural preservation was associated with greater odds of hypertension and subclinical atherosclerosis. However, the association with subclinical atherosclerosis was attenuated after adjustment for hypertension, suggesting that hypertension may partly account for this finding. By integrating sociocultural indicators with clinical and vascular measures, this study highlights the importance of considering cultural context when characterizing cardiovascular risk in populations with a longstanding cultural heritage.

## *Supplemental Materials

For additional information, please click here. https://abccardiol.org/supplementary-material/2026/12310/2026-0095_Supplementary.pdf
